# SPIRALS: An Approach to Non-Linear Thinking for Medical Students in the Emergency Department

**DOI:** 10.7759/cureus.9727

**Published:** 2020-08-13

**Authors:** Rebecca N Small, Lisa Fleet, Desmond Whalen, Tia S Renouf

**Affiliations:** 1 Internal Medicine, Memorial University of Newfoundland, St. John's, CAN; 2 Office of Professional Development, Memorial University of Newfoundland, St. John's, CAN; 3 Emergency Medicine, Memorial University of Newfoundland, St. John's, CAN

**Keywords:** medical education, undergraduate, emergency medicine, delphi, medical students, competencies, clinical reasoning

## Abstract

Context

We lack guidelines to inform the necessary components of an emergency medicine undergraduate rotation. Traditionally, clinical reasoning has been taught using linear thought processes likely not ideal for diagnostic and management decisions made in the emergency department.

Methods

We used the Delphi method to obtain consensus on a set of competencies for undergraduate emergency medicine that illustrate the non-linear concepts we believe are necessary for learners. Competencies were informed by a naturalistic observational study of emergency physicians. A survey outlining these competencies was subsequently circulated to emergency physicians who rated their relative importance.

Results

Eleven competencies were included in Round 1, all rated within the “for consideration” for inclusion range. This was reduced to 10 competencies in Round 2, which was only marginally more definitive with respondents rating one competency in the “definite inclusion range” and the remaining in the “for consideration” range.

Conclusions

This study was conducted to address a gap in the current undergraduate emergency medicine curriculum. Consensus on the relative importance of each competency was not achieved, though we believe that the competencies that arose from this study will help medical students develop the non-linear thinking processes necessary to succeed in emergency medicine.

## Introduction

Historically, we have lacked undergraduate guidelines and standards across several medical fields [1]. While both the Royal College of Physicians and Surgeons of Canada (RCPSC) and the College of Family Physicians of Canada (CFPC) define objectives and competencies for postgraduate trainees in emergency medicine, the foundation for this advanced training begins during medical school [1,2]. 

In Canada, the RCPSC refined its guidelines and standards with a competency-based framework called CanMEDS in 1996. Updates in 2005 and 2015 label the component roles as medical expert, communicator, collaborator, leader, health advocate, scholar, and professional. In 2009, the CFPC adopted similar roles, with each also linked to a set of competencies [2]. These competencies were traditionally taught in linear thought processes which may not be ideal for the Emergency Department (ED) where undergraduates must constantly evaluate and re-evaluate their assessments, diagnoses, and management plans based on changes in clinical status and response to clinical interventions. This practice is essential as fully trained emergency practitioners (EPs) must make rapid diagnostic and management decisions for undifferentiated patients, often with very little clinical information available and while under severe time constraints with a high cognitive load. Linear thinking would therefore seem to leave the learner several steps behind when addressing these complex and evolving clinical situations. 

In 2013, Penciner et al. addressed this gap by using the Delphi method to create a consensus on core undergraduate medical competencies [3]. Similarly to Penciner et al., we used the Delphi method to further develop such competencies. We applied our work to several medical education theories in order to help develop not only competent, but also flexible EPs, as we argue that flexibility is the key to success in this field [4].

In earlier work, we developed the SPIRALS mnemonic (Sick, Pain, Investigate, Resuscitate, Assess (again), LeaveS) in order to guide undergraduates in their approach to the undifferentiated patient in the ED and ultimately facilitate non-linear thinking. In this paper, the Delphi method was used to achieve consensus on the non-linear concepts and skills that we suggest are necessary for learners to grasp within the ED setting. We addressed one potential theoretical basis for non-linear thinking, Durning et al.’s Situativity Theory, as well as Ilgen et al.’s work which explores “comfort with uncertainty,” and Bhat et al.’s work on “threshold concepts” (TC) as they relate to non-linear processes [5,6,7,8].

## Materials and methods

Purpose

**Figure 1:**
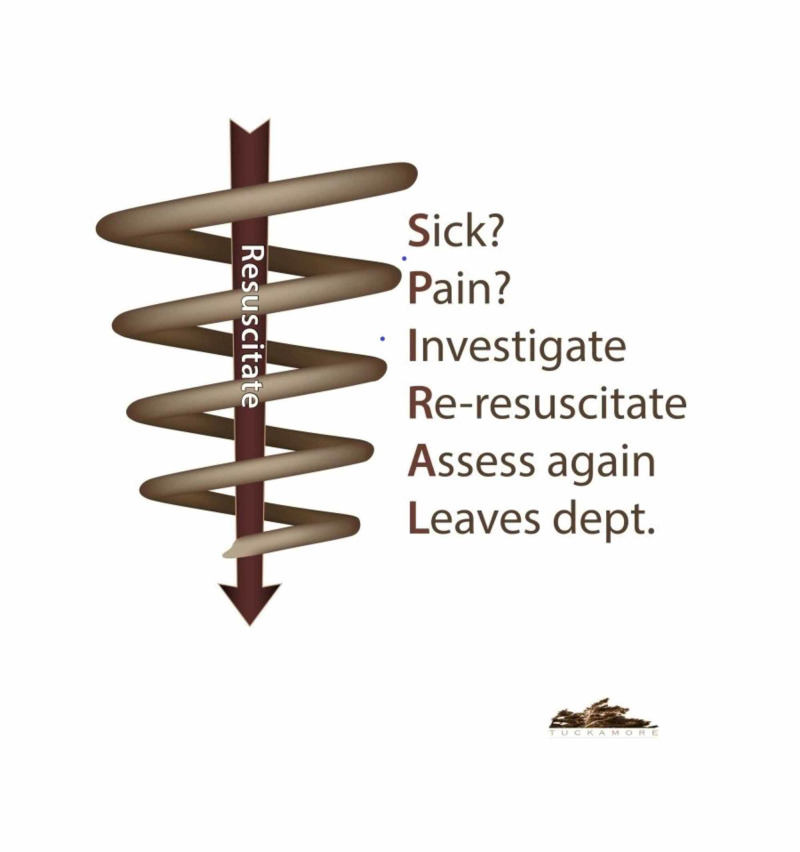
SPIRALS Logo The SPIRALS logo aims to show the central role of resuscitation in this model, and that all other actions will circle back to this central concept [9].

This study used a modified Delphi method to develop a set of core competencies for the undergraduate emergency medicine rotation in a Canadian medical program. The competencies encourage the SPIRALS (non-linear) thinking required of ED physicians. The SPIRALS logo is pictured in Figure 1.

Preliminary naturalistic observation study

**Figure 2:**
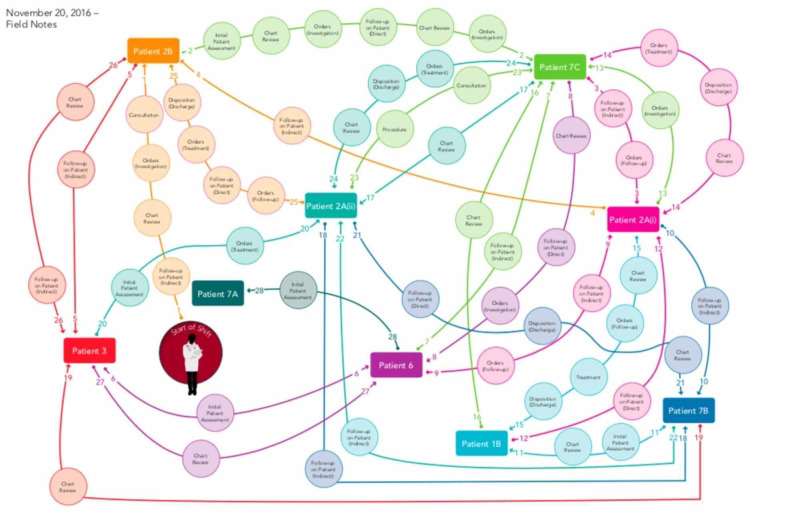
Concept Map Example

We decided that a pilot study was unnecessary given that eight emergency medicine physician-experts contributed to the development of the competencies in the study phase prior to the initiation of the Delphi method-based competency development. Eight EPs were observed by a paired clinical and non-clinical research assistant over the course of their shifts. The clinical researcher provided context, while the non-clinical research assistant blindly grouped tasks under several headings. Following the observation, thematic analysis identified common behaviours by transcribing the notes from each observation and uploading the data into NVivo software [10]. An initial list of themes was created by two team members and refined by a third to ensure consistency with practices in the ED. Using the list of themes, all field notes were reviewed and coded by two team members to generate the final list of themes. The Kappa statistic was used to determine the consistency of coding among team members. A concept map was created for each observation. Each of the EPs observed were invited to review the concept map created depicting their respective observations via questionnaire. Respondents were asked to indicate whether the concept map provided accurately represented their behaviours/activities during their shift. An example of one concept map is seen in Figure 2. 

Round 1 Delphi

Competencies were identified based on the thematic analysis described in the preliminary study above. Both rounds of the survey were reviewed and approved by the Health Research Ethics Authority in Newfoundland and Labrador. The Delphi survey, which included the 11 competencies, was designed using a combination of closed and open-ended items. Respondents’ perceptions of the ED were explored by asking them to use three words to describe a shift in the department. Respondents were then asked to rate the importance of teaching each of the 11 competencies on a 7-point Likert scale, ranging from 1=not important to 7=extremely important, while providing a rationale for their ratings. Demographic data collected from respondents included their years of ED experience, the setting of their experience (teaching hospital, large or small ED), and their current status within the Faculty of Medicine at Memorial University. Physicians and residents working in various EDs across the province were invited to participate in the study via e-mail by the Discipline of Emergency Medicine. The survey was distributed online via Surveymonkey.com® and completion of the survey implied consent. Survey respondents were also required to provide their names and e-mails to ensure Round 2 follow-up. 

Competencies rated as a 6 or 7 were included in Round 2; competencies rated at 4 or 5 would be “for consideration”; and competencies rated 1, 2 or 3 were not included in the Round 2. Team members read through respondents’ comments related to each of the competencies in Round 1 to establish which parts of the competency appeared to be most important to physicians. The comments physicians provided were used to re-write the competencies in a manner more consistent with the physicians’ feedback. Two team members also used their recent experiences as undergraduates to help establish realistic and useful competencies. 

Round 2 Delphi

Modified competencies were re-distributed to physician experts in Round 2. As in Round 1, the survey URL was distributed via e-mail with the survey posted online using Surveymonkey.com®. Round 2 consisted of 10 competencies. Respondents were presented with the original Round 1 competency (including its Delphi mean score) then asked to rate the revised competency in comparison and provide a rationale for its rating. 

## Results

Round 1 Delphi

**Figure 3:**
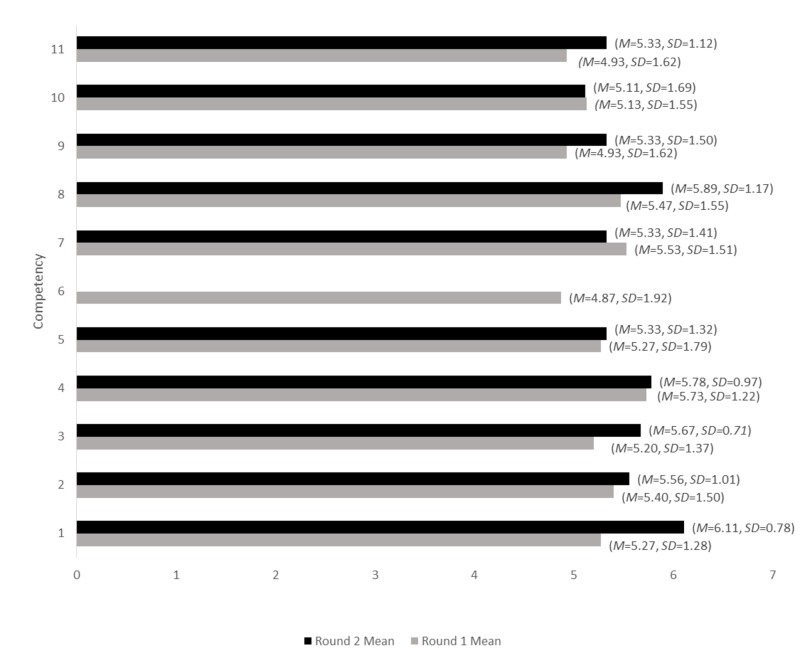
Comparison of the mean rating of each competency between rounds

Competencies were discussed by 15 physician-experts: nine full-time faculty, five part-time faculty, and one resident. Ten of the respondents had worked in the ED for more than five years, three had worked in the ED for three to five years, and two had worked in the ED for one to three years. Eleven respondents had worked only in Category A EDs (24-hour on-site emergency coverage), while four respondents had worked in both Category A and Category B (24-hour on-call service) departments [11]. The mean rating for each competency in each round is seen in Figure 3.

Round 1 qualitative data by competency

1. Develop and maintain a working plan of three to five patients under your care, from arrival in ED to disposition from ED.

Some respondents felt the development of a plan was beyond the level of an undergraduate, though one felt this was important as “that’s basically the whole job.” Some described three to five patients as “ideal,” teaching learners to multi-task, while others felt this quantity would compromise quality. 

2. Independently initiate, repeatedly reassess, and act upon the patients’ resuscitation needs.

Several respondents deemed this competency as beyond the level of an undergraduate, though they recognized the importance of re-assessments. One respondent commented that learners often get “caught up” with initial assessments and forget to re-assess how patients are responding to interventions.

3. Independently initiate, repeatedly reassess, and act upon the patients’ analgesia needs.

Several respondents emphasized the undergraduate’s need to learn to recognize and assess pain, though they will not yet order medications independently at this stage.

4. Independently initiate, repeat, and act upon effective communications with interdisciplinary team members.

Several respondents noted the importance of communicating not only with physicians and allied health, but also with consulting services. Two respondents commented on the need for efficiency in order to “weed out” extraneous details, while another suggested that while important, speaking with consulting services is difficult for some undergraduates who should not be pushed to do something they are not yet prepared for or comfortable with.

5. Continually retrieve patient test results or ask an interdisciplinary team member to do so.

Respondents felt this was appropriate for undergraduates to do independently, though they should also critically evaluate the impact of tests on diagnosis and management within the context of limited hospital resources. One respondent didn’t feel this was an essential competency as this should be automated in the ED.

6. Ensure patient test results are in hand in a timely fashion.

Five respondents suggest this competency is a “reasonable expectation” for undergraduates, while one reported that this should be automated in the ED.

7. Independently and repeatedly assess patient responses to interventions or ask an interdisciplinary team member to do so.

Several respondents commented that this competency is already encompassed in other competencies, though still important.

8. Change patient diagnosis and/or management and disposition (if necessary) according to ongoing patient re-assessment.

Several respondents commented on the importance of not fixating on an initial plan and developing the flexibility to adapt to changing circumstances, but felt this would likely require a fairly high degree of supervision.

9. Independently and repeatedly assess and (if necessary) modify planned tests/treatments/diagnosis according to the patient's response to interventions already performed.

Respondents felt this competency was beyond the level of an undergraduate, but should be learning these skills with close supervision.

10. Independently and repeatedly communicate with patients and family and ask if social and systems-related factors affect their family member’s care. Modify investigations and disposition accordingly.

Respondents commented that the ED “shifts present great opportunities to hone communication skills, especially in stressful situations” and most felt this was a very important competency; however, two respondents felt that undergraduates will still require significant guidance at this stage.

11. Collaborate with the interdisciplinary team to develop an early disposition plan, and (if necessary) modify the plan as new clinical information becomes available.

Respondents felt this competency just reiterated skills that had already been outlined in previous competencies.

Round 2 Delphi

The revised competencies informed by Round 1 were re-distributed in Round 2 to the same physician-experts as in Round 1. The competencies were discussed by nine physician-experts this time; seven were full-time faculty members, two part-time faculty members and no residents responded in this round. Seven respondents had worked in the ED for more than five years, one for three to five years and one for one to three years. Six respondents had worked only in Category A EDs, while three respondents had worked in both Category A and Category B departments.

Round 2 qualitative data

1. Develop and maintain a working plan for two to three patients under your care, from arrival in, to disposition from, ED. 

Two respondents felt this was too many, though, one re-iterated the need for multi-tasking skills.

2. Be able to recognize, repeatedly re-assess, plan interventions, and follow-up on patients’ resuscitation needs. 

Respondents felt that this was above the level of a clinical clerk.

3. Be able to recognize a patient’s analgesia needs and suggest an analgesia management plan to staff.

One respondent commented that the wording of this competency was much more appropriate to clerkship training than the original competency proposed in Round 1.

4. Ongoing communication with interdisciplinary team members and act upon recommendations from team members.

Respondents preferred this wording over Round 1.

5. Independently retrieve patient test results in a timely manner and re-assess as appropriate. 

Respondents preferred this wording over Round 1.

6. Removed after review in advance of Round 2, as it was integrated into competency 5.

7. Repeatedly assess patient responses to interventions or ask an interdisciplinary team member to do so. 

One respondent highlighted the importance of undergraduates making a habit of re-assessing patients rather than relying on nurses to do so.

8. Consider a broad differential diagnosis and associated management/disposition and recognize that these may change during continual patient re-assessment.

One respondent felt this wording was appropriate to the undergraduate level while another felt it was of a higher level than most clerks would obtain.

9. Repeatedly assess and (if necessary) suggest modifications to planned tests/treatments/diagnosis according to the patient's response to interventions already performed. 

One respondent felt this wording was improved over Round 1, while another felt this was still beyond the ability of most undergraduates.

10. Communicate with patients and family and ask if social and systems-related factors affect their family member’s care. Suggest modifications to investigations and disposition accordingly. 

One respondent stated that they did not know what the term “systems-related factors” was referring to.

11. Suggest an early disposition plan and modify the plan as new clinical information becomes available.

This wording was preferred over the equivalent Round 1 competency. Comparisons of the mean ratings of each competency are seen in Figure 3.

## Discussion

Some researchers in the field of medical education believe that our long-held focus on diagnosis may actually be detrimental to a learner’s development of clinical reasoning; rather, we should be instilling in learners that problem-defining and problem-solving must exist in parallel rather than in sequence [12,13,14]. This allows learners to use their provisional diagnoses as a framework for action rather than as an absolute. Researchers also recognize that learners often experience difficulty transferring knowledge to new situations if it was learned in another context [15,16]. Situativity Theory supports both of these concepts by arguing that clinical reasoning is likely non-linear and is situated in experience. “From a situated perspective, linearity is likely to occur only in straightforward presentations” [17]. While an educator may provide a learner with a tool (knowledge), the educator must remember to also teach the learner how to use this tool. In contrast to information processing theory, which involves knowledge simply being transferred from a teacher to a learner, Situativity Theory recognizes the importance of the participants, the physical environment in which the learning takes place and the interactions between these entities in the attainment of knowledge [5]. The SPIRALS tool was created in accordance with Durning et al.’s theory of using non-linear thinking patterns to best acquire and re-apply situational knowledge while working in the ED by prompting the learner to constantly re-evaluate their perception of the medical problem, diagnosis and management plan. Learners subsequently develop the flexibility necessary to successfully manage the wide variety of medical situations that characterize emergency medicine.

Ilgen et al. encourage learners to attend to their own perceptions of their cognitive experiences to guide further action. For example, learners may experience “comfort” or “discomfort”; the feeling of “discomfort” may prompt a learner to re-evaluate a situation and recognize the potential for danger, prompting them to seek help and change their course of action [18]. The feeling of “comfort” can act as a check-point, reminding the learner that they are on track, acting as a type of re-evaluation [6]. Ilgen et al. discuss “comfort with uncertainty” in the ED setting [6]. First, learners must be guided away from the traditionally held notion that medical knowledge falls into the binary categories of “knowing” and “not knowing,” but rather a spectrum of “informed speculation,” allowing for more flexibility [19]. In their earlier work, the authors note how over time, through didactic teaching, observation and clinical experience, junior learners form an easily retrievable mental framework for each diagnosis or clinical presentation, just as their expert counterparts already do [7]. The SPIRALS framework attempts to guide learners through this process; for example, the discomfort described above prompts the student to question their decisions, re-evaluate the situation and consider alternative diagnoses and management plans as suggested by competency 9 in our study, “repeatedly assess and (if necessary) suggest modifications to planned tests/treatments/diagnosis according to the patient response to interventions already performed.”

Bhat et al. describe a “threshold concept” (TC) as a “transformative and troublesome concept critical to the transition from trainee to practitioner” [8]. TCs are often irreversible; once experienced, the learner can no longer perceive knowledge as they did before. “Active learning” and “burden of responsibility” are examples of TCs. In the former, learners recognize that managing unfamiliar case presentations creates new knowledge, but not without the risk that the learner could be wrong in diagnostic or management decisions. Using this active learning model, learners must proactively create their own approach rather than wait to be told what to do. While each competency described in our study describes a different aspect of patient care, all competencies are centered upon the principle of developing a plan, while recognizing the need to re-evaluate plans based on new information and evolving clinical situations. This allows trainees to adopt a flexible and active approach to patient management. “Burden of responsibility” is another TC in which trainees recognize their role is significant; however, they may grapple with the responsibility placed upon them [8]. This concept is a key element of the SPIRALS approach; rather than passively collecting information and reporting it to staff, we encourage undergraduates to take an active role in their education by developing their own ideas, plans, and revisions to these plans, taking ownership of the situation, and using staff physicians as teaching mentors. 

Limitations

The flexibility of the Delphi method may come at the expense of challenges to its validity. Judgements on procedure must often be made by researchers in the absence of evidence or good reasons for making certain choices. There are also some challenges to the validity of results. There are several built-in mechanisms within the methodology that attempt to combat these challenges, for example, the ‘safety in numbers’ principle, however, this concept itself confers some inherent pressure to conform to the opinions of others. Finally, another important limitation of the Delphi study design is its lack of consistent methods for analyzing data and reporting results. There is currently no universally accepted level of consensus recommended for Delphi projects or reporting guidelines, further detracting from the rigour of the methodology.

In a study similar to ours, Penciner et al. used emergency medicine educators who did not participate in the study as external reviewers who were asked to comment on whether or not they felt the study results were valid, useful, and applicable to an undergraduate curriculum in emergency medicine. They were also asked to comment on the appropriateness of the methodology used in the study as a means to inform competency and curriculum development. The authors of this study do also speak to the difficulty in validating the Delphi process as there is still active debate in the literature concerning the topic. The authors comment that they attempted to enhance the validity of their study by choosing a representative panel of experts from across Canada, representing all provinces except two. They also noted their effort to recruit experts with diverse backgrounds as well as their high response rate as a strength of the study which enhanced its validity [3].

Finally, another limitation of our study it the lack of validation of the SPIRALS tool itself. We hope to undertake this work in the future.

## Conclusions

We undertook this study in order to address a gap in the current Canadian medical school curriculum by creating a standardized list of objectives and competencies for the undergraduate emergency medicine rotation. Previous research identified some of the unique challenges learners face during their emergency medicine rotation, often revolving around the fact that physicians must make rapid decisions, often in the context of very limited clinical information, a daunting task for most inexperienced trainees. Undergraduates taught to think in a non-linear fashion will be better equipped to manage the often chaotic nature of the ED by developing the flexibility needed to adapt and even thrive in the face of each new challenging clinical encounter.
